# Deciphering the Novel Role of AtMIN7 in Cuticle Formation and Defense against the Bacterial Pathogen Infection

**DOI:** 10.3390/ijms21155547

**Published:** 2020-08-03

**Authors:** Zhenzhen Zhao, Xianpeng Yang, Shiyou Lü, Jiangbo Fan, Stephen Opiyo, Piao Yang, Jack Mangold, David Mackey, Ye Xia

**Affiliations:** 1 Department of Plant Pathology, College of Food, Agricultural, and Environmental Science, The Ohio State University, Columbus, OH 43210, USA; zhao.2047@osu.edu (Z.Z.); opiyo.1@osu.edu (S.O.); yang.4636@osu.edu (P.Y.); mangold.20@buckeyemail.osu.edu (J.M.); 2 College of Life Sciences, Shandong Normal University, Jinan 250014, China; yangxp2006@sdnu.edu.cn; 3 State Key Laboratory of Biocatalysis and Enzyme Engineering, School of Life Sciences, Hubei University, Wuhan 434200, China; shiyoulu@hubu.edu.cn; 4 School of Agriculture and Biology, Shanghai Jiao Tong University, Shanghai 200240, China; fan.0127@yahoo.com; 5 Department of Horticulture and Crop Science, College of Food, Agricultural, and Environmental Science, The Ohio State University, Columbus, OH 43210, USA; mackey.86@osu.edu

**Keywords:** plant cuticle, cutin, vesicle trafficking, transport, plant hormones, plant defense

## Abstract

The cuticle is the outermost layer of plant aerial tissue that interacts with the environment and protects plants against water loss and various biotic and abiotic stresses. ADP ribosylation factor guanine nucleotide exchange factor proteins (ARF-GEFs) are key components of the vesicle trafficking system. Our study discovers that AtMIN7, an *Arabidopsis* ARF-GEF, is critical for cuticle formation and related leaf surface defense against the bacterial pathogen *Pseudomonas syringae* pathovar tomato (*Pto*). Our transmission electron microscopy and scanning electron microscopy studies indicate that the *atmin7* mutant leaves have a thinner cuticular layer, defective stomata structure, and impaired cuticle ledge of stomata compared to the leaves of wild type plants. GC–MS analysis further revealed that the amount of cutin monomers was significantly reduced in *atmin7* mutant plants. Furthermore, the exogenous application of either of three plant hormones—salicylic acid, jasmonic acid, or abscisic acid—enhanced the cuticle formation in *atmin7* mutant leaves and the related defense responses to the bacterial *Pto* infection. Thus, transport of cutin-related components by AtMIN7 may contribute to its impact on cuticle formation and related defense function.

## 1. Introduction

The plant cuticle is the outermost hydrophobic layer of the aerial plant surfaces which functions as a mechanical barrier to provide physical support, avoid organ fusion, and control the surface water status [1,2]. The cuticle, which covers most of the plant tissues, including the leaves, fruits, flowers, and non-woody stems, is the first line to contact the outside environment [3,4,5]. The cuticle protects plants against various abiotic and biotic stresses, including extreme temperatures, drought, UV radiation, and attacks by pathogens and insects. The status of the cuticle varies with the stage of plant development as well as environmental factors. For instance, a pathogen’s entry into the plant tissue is the critical first step during the infection process, which leads to compositional and functional changes of the cuticle. Conversely, pathogens can sense the changes of the plant cuticle and adjust their pathogenesis and virulence accordingly [6,7]. Emerging research indicates that the plant cuticle participates in preemptive plant defense, such as during systemic acquired resistance (SAR) [7,8,9]. However, the regulation and role of the cuticle in plant defense are still poorly understood.

With the development of genetic, molecular, and biochemical approaches, we have a better understanding of the components and mechanisms involved in cuticle biosynthesis and related functions in the past decade. Plant cuticles are mainly composed of cutin and cuticular waxes. In *Arabidopsis*, cutin is a core structural polymer of hydroxy and/or epoxy fatty acids. The cuticular wax covering the cutin matrix is a complex mixture consisting of very long-chain fatty acid (VLCFA) derivatives, including alkanes, aldehydes, primary and secondary alcohols, ketones, and esters [1,3,10]. Cutin and wax biosynthesis both begin with the de novo C16 and C18 fatty acids in the plastid of plant epidermal cells. Plastid-generated C16 and C18 fatty acids are esterified to Co-enzyme A (CoA) to form C16/18-CoA before entering the endoplasmic reticulum (ER). In ER, the C16/18-CoA is converted either to very long-chain Acyl-CoA, which are precursors for wax biosynthesis, or to 16-OH C16/18-CoA, which are precursors for cutin biosynthesis. The wax and cutin precursors must be further modified and exported from the ER, move across the plasma membrane (PM) and the polysaccharide cell wall, and ultimately self-assemble and deposit into the cuticle layer [3,10,11].

The transport of cutin- and wax-related components can be divided into intracellular (translocation from the ER to the PM) and extracellular (translocation through the cell wall to the cuticle layer) trafficking [1,3]. A variety of mechanisms have been suggested for the intracellular transport of wax and cutin components, including the direct physical ER–PM contacts; transport of wax- and cutin-related components by soluble cytosolic proteins, such as LTPs (lipid transfer proteins) and fatty acid binding proteins; self-assembly to form oleophilic droplets that bud out of the ER to PM; and a Golgi or trans-Golgi network-mediated secretory pathway [1,12,13,14,15,16]. The export of the cuticular component translocation across the PM to the extracellular space is predicted to be carried out by ATP-binding cassette (ABC) transporters and LTPs [13,17]. For instance, CER5, also known as ABCG12, is required for wax export in *Arabidopsis* [18]. ABCG11 is required for both cutin and wax extracellular export [19]. Another transporter, ABCG13, is critical for cutin deposition in *Arabidopsis* flowers [20,21]. Glycosylphosphatidylinositol-anchored lipid transfer proteins 1 and 2 (LTPG1 and LTPG2), which bind to a variety of lipid substances, were found to participate in the export of wax-related components [22]. Still, the mechanisms of transporting cuticle-related components need to be further investigated.

Vesicle trafficking, which mediates the transport of materials between different cellular compartments and between a cell and its environment, is critical for plant growth, development, and responses to the environmental factors, including the defense against pathogen infections [23,24]. There are two categories of trafficking at the plasma membrane: exocytosis secretes vesicle contents to the extracellular space, and endocytosis imports extracellular materials into intracellular vesicles [23]. Upon interactions with the pathogenic microbes, endocytosis regulates the activity of surface-localized immune receptors [25,26]. Meanwhile, the components for cell wall fortification, antimicrobial compounds, and defense proteins are delivered to the pathogen invasion sites through polarized exocytosis [24,27]. In plants, the trans-Golgi network/early endosome (TGN/EE), which serves as a key sorting station at the intersection of exocytic and endocytic pathways, plays a critical role in sorting and delivery of the soluble defense proteins to the extracellular space or the membrane localized cargo to PM during the immune activation [23,28]. Several important components of TGN/EE are involved in the trafficking of specific immune-related cargoes [29]. For instance, the TGN/EE-localized E3 ubiquitin ligase Keep On Going plays a critical role in sorting and delivering of the pathogenesis-related protein 1 and papain-like Cys protease C14 to the extracellular space [30]. The TGN/EE-resident SNARE protein VAMP721/722 was found to regulate the delivery of RPW8.2, a key protein in resistance against the powdery mildew pathogen, to the extrahaustorial membrane, which is the fungal pathogen feeding structure [31]. Clathrin-coated vesicles (CCVs) contribute to immune cargo trafficking, which regulates the plant immune responses [32]. However, little is known about the defense-associated trafficking of cuticular wax and cutin components to the cuticle layer.

ADP ribosylation factor guanine nucleotide exchange factors (ARF-GEFs) are the key components of the vesicle trafficking system [33,34]. AtMIN7, one of eight ARF-GEFs in *Arabidopsis*, is localized to the TGN/EE where it regulates the plant resistance against *Pseudomonas syringae* pathovar tomato DC3000 (*Pto* DC3000) [35,36,37,38]. ARF proteins switch between the active (GTP-bound) and inactive (GDP-bound) states. Upon activation, ARFs bind to membranes and promote vesicle budding at the TGN, endosomal compartments, and PM [39,40]. Highlighting the importance of AtMIN7 for plant immunity, the HopM1 virulence effector from *Pto* DC3000 contributes to bacterial virulence by interacting with and triggering proteasome-mediated degradation of AtMIN7 [35,36].

In this study, we found that *atmin7* mutant plants are defective in cuticle formation by toluidine blue staining and direct observation with transmission electron microscopy (TEM) and scanning electron microscopy (SEM). Consistent with these observations, GC–MS analysis showed that cutin monomer contents were specifically reduced in mutant leaves. Furthermore, it was discovered that the exogenous application to *atmin7* mutant leaves of either of three plant hormones—salicylic acid (SA), jasmonic acid (JA), or abscisic acid (ABA)—could enhance the cuticle formation and restore leaf surface defense responses against *Pto* DC3000. Taken together, our results indicate that AtMIN7 is critical for cutin formation, perhaps by supporting the transport of precursor subunits and/or related enzymes. Furthermore, AtMIN7 may integrate the response to hormones with modification of the cuticle for the defense against *Pto* DC3000 infection in *Arabidopsis*.

## 2. Results

### 2.1. Molecular Perturbation of AtMIN7

**Figure 1:**
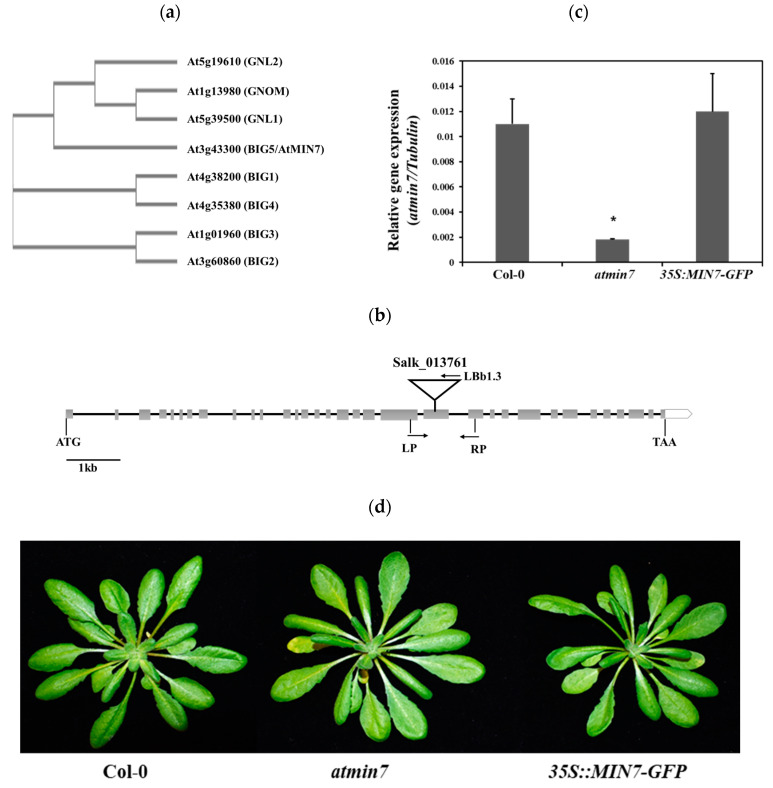
Characterization of the *atmin7* mutant. (**a**). Phylogenetic analysis of the ARF-GFF protein family in *Arabidopsis*. (**b**). Schematic representation of the T-DNA insertion site in *atmin7* mutant. Heavy black lines and gray rectangles indicate introns and exons, respectively. The T-DNA insertion site for *Salk_013761* is shown. The position and orientation of primers used for qRT-PCR are indicated. (**c**). Relative *AtMIN7* transcript abundance in the WT, *atmin7*, and *35S::MIN7-GFP* plants. The experiment was repeated three times with similar results and the mean +/- SD for the combined data from three replicates are shown. Unpaired two-tailed Student’s *t*-tests with WT plants indicated that the transcript level of *AtMIN7* was significantly reduced in *atmin7* but not *35S::MIN7-GFP* (* *p* < 0.01). (**d**). Morphological phenotype did not differ between vegetative rosettes of four-week-old WT, *atmin7*, and *35S::MIN7-GFP* plants.

*Arabidopsis* encodes eight ARF-GEFs, each of which contains a Brefeldin A (BFA)-inhibited Sec7 catalytic domain that activates ARF proteins by exchanging GDP for GTP [40]. These are divided into two subfamilies: the GGG class which includes GNOM, GNOM-LIKE1(GNL1), and GNL2; and the BIG (BFA-inhibited guanine nucleotide exchange proteins) class which includes five members (BIG1-5) [39]. To investigate their relationships, a multiple protein sequence alignment of all eight ARF-GEFs isoforms was created using Clustal Omega (https://www.ebi.ac.uk/Tools/msa/clustalo/). AtMIN7/BIG5 does not cluster with the other four BIG isoforms (Figure 1a), which indicates that it might have a unique function.

The T-DNA insertion line of *AtMIN7* (*AT3G43300*) (Salk_013761) was obtained from the Arabidopsis Biological Resource Center in USA and the homozygous line was identified based on the protocol developed by the Salk Institute Genomic Analysis Laboratory (http://signal.salk.edu/tdnaprimers.2.html). This mutant line, designated as *atmin7* [35], has a T-DNA insertion in the 21st exon (Figure 1b). The abundance of the *AtMIN7* transcript was much lower in *atmin7* than in WT Col-0 plants (Figure 1c). To further confirm the function of *AtMIN7*, an *atmin7* transgenic complementation line with a transgene containing the whole cDNA sequence of *AtMIN7* driven by a 35S promoter (*35S::MIN7-GFP*) was obtained from Dr. Shengyang He’s lab [35,36]. The transcript level of *AtMIN7* in the complementation line was similar to that of WT plants (Figure 1c). According to the *Arabidopsis* eFP Browser (http://bar.utoronto.ca/eplant/), *AtMIN7* is constitutively and highly expressed in stems, leaves, roots, and flowers (Figure S1). Macroscopic examination did not reveal any differences between the *atmin7* mutant, complementation line, and WT plants (Figure 1d).

### 2.2. A Mutation in AtMIN7 Results in the Defective Cutin Layer and Stomata Ledge

**Figure 2:**
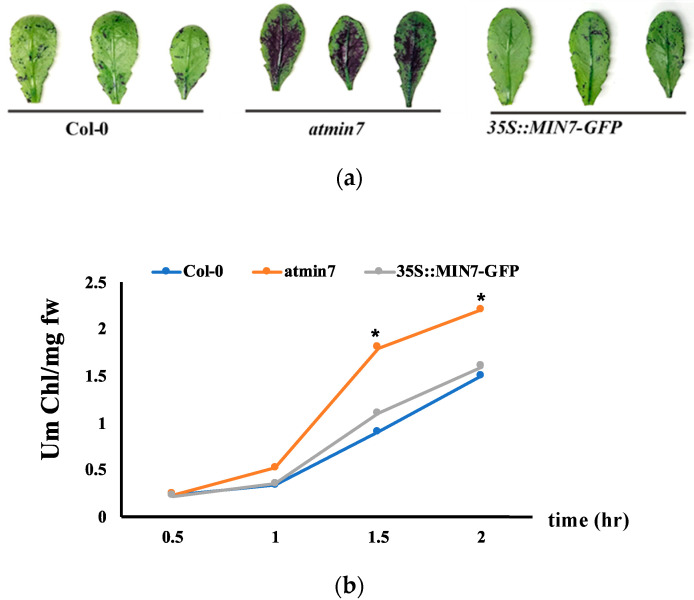
*AtMIN7* is required for the normal cuticle formation. (**a**). Leaves of four-week-old WT Col-0, *atmin7*, and *35S::MIN7-GFP* complementation plants stained with TB. The mutant *atmin7* plants showed the TB staining but not the WT and *35S::MIN7-GFP* complementation plants, which indicated the defective cuticle in *atmin7*mutant plants. (**b**). Chlorophyll leaching rate of the leaves of WT (blue circles), *atmin7* (orange circles), and *35S::MIN7-GFP* (grey circles) plants. The experiment was repeated three times with the similar results and mean +/- SD for the combined data from three replicates are shown. Unpaired two-tailed Student’s *t*-tests with WT plants indicated that chlorophyll leaching did not differ from *35S::MIN7-GFP* but was significantly increased at 1.5 and 2 h in *atmin7* plants (* *p* < 0.05), which indicated the defective cuticle in the *atmin7* mutant plants.

Toluidine blue (TB) is a hydrophilic dye that more efficiently penetrates leaves with a defective cuticle [41]. TB staining of four-week-old WT, *atmin7*, and *35S::MIN7-GFP* plants indicated a defective cuticle in the leaves of *atmin7* plants (Figure 2a). A defective cuticle results in faster chlorophyll leaching in 80% ethanol [9,42]. Consistent with the results of TB staining, chlorophyll leached faster from the *atmin7* leaves compared to those of the WT and complementation line (Figure 2b).

**Figure 3:**
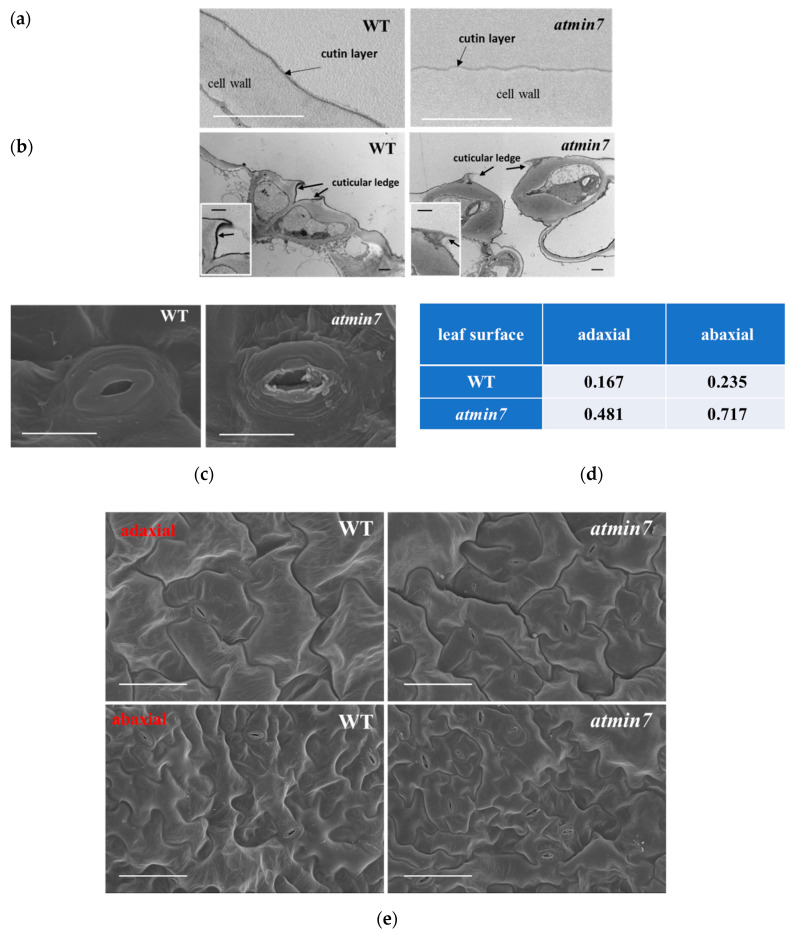
TEM and SEM reveal the defective cuticle and stomatal structure in the leaves of *atmin7* plants. (**a**). TEM images of the cutin layer (indicated by black arrows) on adaxial surfaces of WT and *atmin7* leaves (scale bars = 1 µm). (**b**). TEM images of the transdermal sections of leaf guard cells. Black arrows indicate the cuticular ledges (scale bars = 1 µm). Insets are magnified images of the cuticular ledges (scale bars = 1 µm). (**c**). SEM images of stomata from abaxial surface of leaves from WT and *atmin7* mutant plants (scale bars = 10 µm). (**d**). Frequency of defective stomata on WT and *atmin7* leaf surface (adaxial and abaxial) detected by SEM (randomly selected 60 stomata for each group). (**e**). SEM images of adaxial and abaxial leaf surface of WT and *atmin7* leaves (scale bars = 50 µm).

Further analysis by TEM indicated that the cuticle layer of *atmin7* was much thinner than the WT plants (Figure 3a). The cuticular ledge is the outermost layer of the stomata pore, which regulates stomatal function [43,44]. TEM also demonstrated that the cuticular ledge of stomata in *atmin7* leaves was almost missing (Figure 3b). SEM was also used to compare the leaf surface of *atmin7* and WT plants. The overall structure of stomata in the leaves of *atmin7* plants, especially the guard cells, was defective compared to those in WT plants (Figure 3c). Randomly selected stomata on both the adaxial (upper) and abaxial (lower) sides of leaves showed the defective structure approximately three times more frequently in *atmin7* than in WT plants (Figure 3d). Pavement cells of the adaxial surfaces of the WT leaves showed the regular puzzle shape while those of *atmin7* leaves contained irregular lobes (Figure 3e). Similarly, epidermal cells on the abaxial surface of *atmin7* leaves were less interconnected relative to the pattern in WT leaves. Furthermore, observation of leaf cross-sections by SEM revealed that the epidermal cells were collapsed in *atmin7* relative to WT plants (Figure S2). Altogether, a mutation in *AtMIN7* resulted in various defects of the plant leaf surface, especially the cuticle of leaf surfaces and the cuticular ledge of stomata.

### 2.3. The Abundance of Cutin Monomers Is Reduced in the Atmin7 Mutant Plants

**Figure 4:**
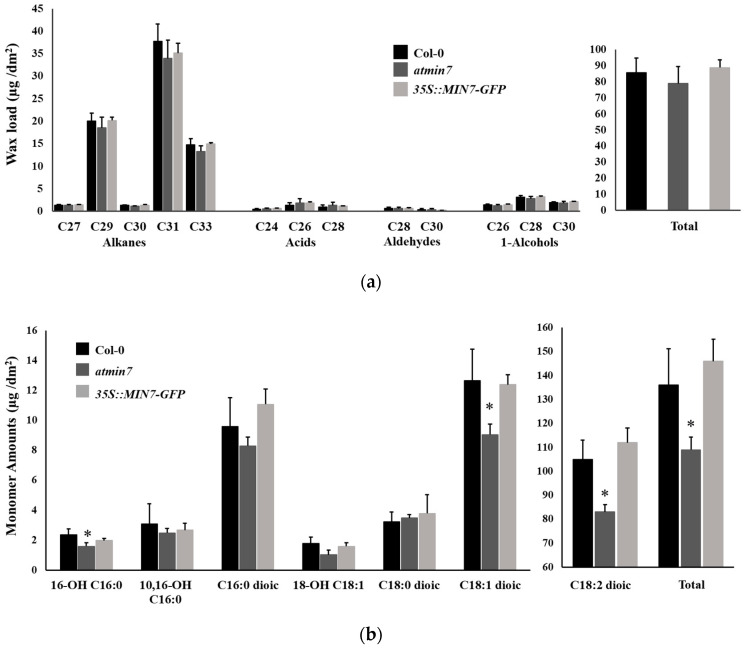
The content of cutin monomers, but not cuticular wax, is abnormal in the leaves of *atmin7* mutant plants. (**a**). Wax load with different chain lengths and total content in the leaves of WT, *atmin7*, and *35S::MIN7-GFP* plants are shown as mean +/- SD. Unpaired two-tailed Student’s *t*-tests with WT did not show significant differences to *atmin7* or *35S::MIN7-GFP* plants (* *p* < 0.05). (**b**). The abundance of cutin monomers and total content in the leaves of WT, *atmin7*, and *35S::MIN7-GFP* plants are shown as mean +/- SD. The C16 and C18 labels on the *x*-axis represent the 16- and 18-carbon acid chains, respectively, whereas the number preceding “OH” indicates the chain insertion point(s). Dioic represents dioic acid. Unpaired two-tailed Student’s *t*-tests with WT show significant differences versus *atmin7* and complementation line plants (* *p* < 0.05).

In addition to the direct observations of the cuticle layer, the leaf cuticular wax and cutin monomer contents were measured by GC–MS. The total leaf cuticular wax load and wax compositions of leaves of *atmin7* did not differ significantly from those of WT plants (Figure 4a). Analysis of the chemical composition of cutin monomers revealed significant reductions in the total levels of cutin monomers in the leaves of *atmin7* relative to those of WT plants, including the major cutin monomer, C18:2 dioic acid, as well as two less abundant cutin monomers, 16-OH-C16:0 acid and C18:1 dioic acid (Figure 4b). The wax compositions and levels of cutin monomers did not significantly differ between WT plants and the complementation line (Figure 4a,b). The significant decrease in the total cutin monomers, including several cutin monomer species, is consistent with the altered ultrastructure of the whole cuticle layer and the cuticular ledge of the stomatal pores in the *atmin7* leaves. Therefore, the mutation of *AtMIN7* significantly influences cutin, but not wax, formation in *Arabidopsis* leaves.

### 2.4. The Atmin7 Mutant Plants Display Increased Susceptibility Following the Bacterial Infection of the Leaf Surface

**Figure 5:**
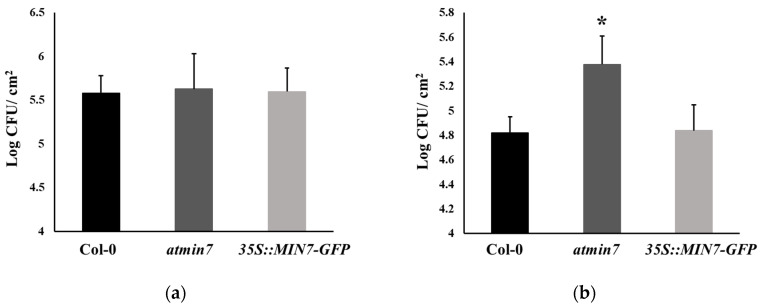
The *atmin7* mutant plants are more susceptible to the *Pto* DC3000 infection on the leaf surface by the spray treatment but not the syringe infiltration. (**a**). Bacterial multiplication in the leaves of WT, *atmin7*, and *35S::MIN7-GFP* plants following the syringe infiltration with 1 × 10^5^ CFU/mL of *Pto* DC3000. (**b**). Bacterial multiplication in the leaves of WT, *atmin7*, and *35S::MIN7-GFP* plants following the surface spray with 1 × 10^8^ CFU/mL of *Pto* DC3000. Following each inoculation method, bacteria were enumerated three days after the infection. The experiments were repeated three times with similar results, and the mean +/- SD for the combined data from three biological replicates are shown. Unpaired two-tailed Student’s *t*-tests with WT plants only revealed a significant difference with *atmin7* following the *Pto* DC3000 infection by surface spray (* *p* < 0.05).

Whether the defective cuticle of *atmin7* mutant plants alters the defense against the infection with *Pto* was investigated. WT, *atmin7*, and *35S::MIN7-GFP* plants were inoculated with *Pto* DC3000 bacterial cells via either of two methods, infiltration with a needleless syringe or surface spray, and the bacterial growth was assessed three days later. The growth of *Pto* DC3000 did not differ between WT and *atmin7* or *35S::MIN7-GFP* plants following the syringe infiltration (Figure 5a). However, the *atmin7* mutant was more susceptible than WT and *35S::MIN7-GFP* when plants were spray-inoculated (Figure 5b). The enhanced susceptibility of *atmin7* following the infection of *Pto* initiating on the leaf surface, but not the leaf interior, is consistent with a role of AtMIN7-dependent stomata and cuticle structure in leaf surface defense [45].

### 2.5. Plant Hormones Rectify the Cuticle Formation and Defense Phenotypes in Atmin7 Mutant Plants

**Figure 6:**
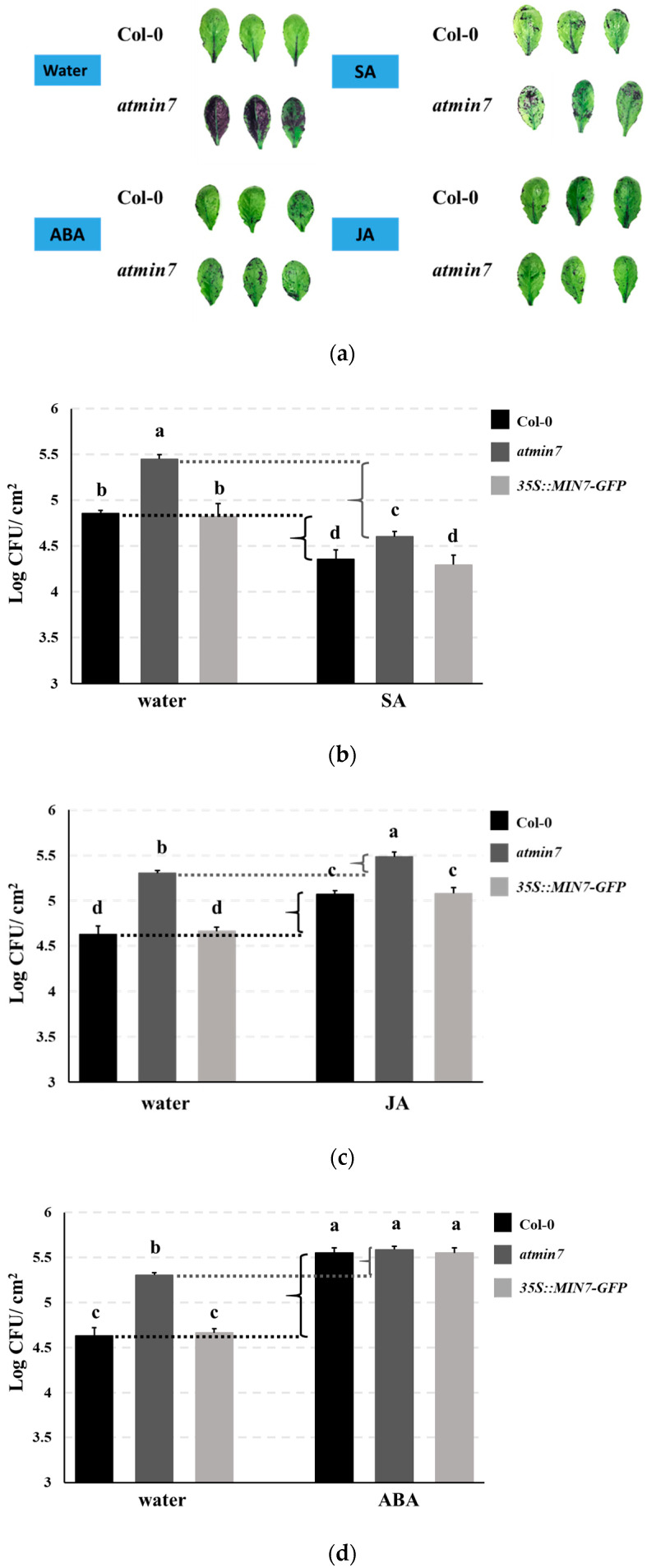
The plant hormones SA, JA, and ABA rescue cuticle formation and defense against the surface infection with *Pto* DC3000 in *atmin7* mutant plants. (**a**). Leaves of four-week-old WT or *atmin7* plants were stained with TB 24 h after the spray treatment with water, SA (1mM), JA (50 µM), ABA (50 µM). 100% EtOH (500 µL) was firstly used to dissolve SA, JA, and ABA, which were further diluted with 1L distilled and deionized water to reach the final treatment concentration, respectively. Accordingly, 1L distilled and deionized water with an equivalent amount of 100% EtOH (500 µL) was sprayed as the control. (**b**–**d**). WT, *atmin7*, and *35S::MIN7-GFP* plants were sprayed with water control and either SA (**b**), JA (**c**), or ABA (**d**) as in the panel. 24 h later, the plant leaves were spray inoculated with 1 × 10^8^ CFU/mL of *Pto* DC3000. Three days later, the bacteria were enumerated. The experiment was repeated twice with similar results, and the mean +/- SD of combined data from the two biological replicates are shown. Different letters a–d within figure (**b**–**d**) indicate the significant differences at *p* < 0.05, which was calculated by one-way analysis of variance (ANOVA) using SPSS ver. 21.

Plant hormones regulate plant growth and development processes, such as cell division, elongation, differentiation, and pattern formation, as well as plant responses to biotic and abiotic stresses [46,47]. Previous studies highlighted the possibility that plant hormone influence cuticle formation [7,48,49,50]. Here, *atmin7* and WT plants were sprayed with different plant hormones [SA (1 mM), JA (50 µM), ABA (50 µM), gibberellic acid (GA_3_, 100 µM), and indole-3-acetic acid (IAA, 10µM)]. 24 h later, the leaves were stained with TB. Application of SA, JA, or ABA, but not water, GA_3_ or IAA, restored the resistance to TB staining in *atmin7* plants, indicating that SA, JA, and ABA could rescue the cuticle formation within one day of their application to *atmin7* plants (Figure 6a and Figure S3).

We further investigated if the enhanced cuticle formation by SA, JA, and ABA could increase the resistance of *atmin7* mutant plants following the surface inoculation with *Pto* DC3000. Plant leaves were sprayed with SA (1 mM), JA (50 µM), or ABA (50 µM) and 24 h later, they were spray-inoculated with *Pto* DC3000. The bacterial growth was then measured three days later. *Arabidopsis* plants upregulate SA signaling and repress JA signaling during the successful defense against hemi-biotrophic strains of *Pto* [45]. Similar to JA, ABA also increases the susceptibility of the plants to *Pto* infection [45,51]. As expected, we observed that treatment with SA increased and treatment with JA or ABA decreased the resistance of WT and *35S::MIN7-GFP* plants to the *Pto* DC3000 infection (Figure 6b–d). In *atmin7* mutant plants, SA and ABA treatments also increased or decreased, respectively, plant resistance to *Pto* DC3000, while an effect of JA was not observed (Figure 6b–d). Notably, the magnitude of enhanced susceptibility of *atmin7* plants, relative to WT and *35S::MIN7-GFP* plants, was reduced following the treatments with SA or JA, and was no longer significant following the treatment with ABA (Figure 6b–d). This result is consistent with the hypothesis that restoration of the cuticle by these plant hormones concomitantly restores leaf surface defense against *Pto* DC3000.

**Figure 7:**
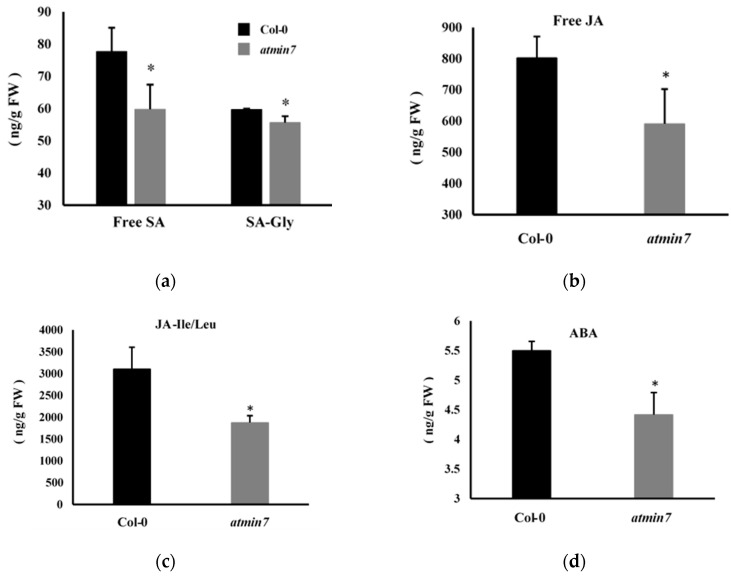
The *atmin7* mutant plants accumulated lower levels of salicylic acid (SA), jasmonic acid (JA), and abscisic acid (ABA) relative to WT Col-0 plants. The levels of free SA and SA-Gly (**a**), free JA (**b**), JA-Ile/Leu (JA-isoleucine/leucine) (**c**), and ABA (**d**) were quantified in the leaves of four-week-old WT and *atmin7* plants. The experiment was repeated twice with similar results, and the mean +/- SD from the two biological replicates are shown. Significant differences between WT and *atmin7* plants were determined by Student’s *t*-test (* *p* < 0.05). FW indicates the fresh weight.

We further determined whether the mutation of *AtMIN7* influenced the levels of SA, JA, and ABA. The quantification of free SA, SA-Gly (an inactive glycosylated derivative of salicylic acid), free JA, biologically active conjugated JA (JA-Ile/Leu), and ABA revealed that the leaves of *atmin7* mutant plants contained significantly lower levels of free SA and JA, JA-Ile/Leu, and ABA compared to the WT plants (Figure 7a–d). Thus, the ability of these hormones to restore the cuticle formation in the *atmin7* mutant may be related to their constitutively low levels in the leaves of the *atmin7* mutant, relative to WT plants.

### 2.6. Transcriptome Analysis of the Atmin7 Mutant Plants

We conducted RNA-Seq to determine the difference in the transcriptome between the leaves of four-week-old *atmin7* and WT plants [2]. A *p*-value of < 0.05 and |log2 fold change (FC)| ≥ 2 were used as the stringent cutoff criteria to designate genes as differentially expressed (DEGs). Based on the criteria, 198 DEGs were downregulated in *atmin7* compared to WT plants (Supplemental Table S1). Given its contribution to cuticle formation, we hypothesize that AtMIN7 mediates the trafficking of cutin building blocks or key enzymes involved in cutin biosynthesis. In addition to this putative direct role of AtMIN7 in cutin formation, many genes hypothetically involved in cutin biosynthesis were downregulated in *atmin7* (Table S2). The cutin precursor used for the extracellular polymerization is thought to be a monoacylglycerol (MAG), likely a 2-MAG [52]. Glycerol-3-phosphate-acyltransferases (GPAT) are involved in the biosynthesis of 2-MAG [53]. Specifically, GPAT4 and GPAT6 catalyze the acyl-transfer or hydrolase reactions important for cutin biosynthesis [54,55]. Notably, *GPAT5* and *GPAT7* were significantly downregulated relative to WT in *atmin7* plants (AT3G11430, 3.80-fold; AT5G06090, 4.68-fold), which might be related to the inhibition of cutin biosynthesis. GDSL-lipases (Gly-Asp-Ser-Leu family of esterases/acylhydrolases) are also required for the formation of cutin polymers [56]. Genes hypothetically encoding two GDSL-lipases were downregulated relative to WT in *atmin7* plants (AT3G50400, 5.68-fold; AT2G23540, 3.00-fold). ABCGs are critical components for the extracellular transport of cuticular wax and cutin from the ER to the cell wall and cuticular layer [21,57,58], and *ABCG6* was downregulated relative to WT in *atmin7* plants (AT5G13580, 2.13-fold). Transcription factors of MYB family are known to regulate wax and cutin biosynthesis in response to biotic and abiotic stresses [59,60,61,62,63,64]. MYB96 and MYB94 were identified to be involved in the activation of wax biosynthesis by drought or ABA treatment [60,63]. MYB30 modulated the plant resistance to avirulent *Pto* strains infection by affecting the very long-chain fatty acid (VLCFA) and VLCFA derivatives (wax components) biosynthesis [59,61]. MYB106, together with MYB16, participates in the cutin biosynthesis in *Arabidopsis* [62]. *MYB41* could be induced by drought, ABA, and salt stress. Overexpression of *MYB41* in *Arabidopsis* plants lead to the downregulation of cutin biosynthesis related genes expression and defective cuticle structure [64]. Our transcriptome data revealed that several *MYB* transcription factors genes were significantly downregulated relative to WT in *atmin7* plants (*MYB41*, AT4G28110, 4.42-fold; *MYB114*, AT1G66380, 4.67-fold; *MYB49*, AT5G54230, 4.42-fold; *MYB17*, AT3G61250, 2.80-fold; *MYB75*, AT1G56650, 2.29-fold), suggesting their possible role in the regulation of cuticle biosynthesis. Lipid transfer proteins (LTPs) and glycosylphosphatidylinositol-anchored lipid transfer proteins (LTPGs) are essential components predicted to transfer lipids or cuticle precursors to their expected destinations [65,66]. Several *LTP* and *LTPG* genes were significantly downregulated relative to WT in *atmin7* mutant (*LTP4*, AT5G59310, 5.44-fold; *LTP3*, AT5G59320, 4.62-fold; *LTP6*, AT3G08770, 3.19-fold; *LTP2*, AT2G38530, 3.12-fold; *LTPG15*, AT2G48130, 3.50-fold; *LTPG5*, AT3G22600, 3.02-fold). Additionally, AT5G41040, which encodes a feruloyl-CoA transferase required for the suberin synthesis, was also downregulated in *atmin7* (AT5G41040, 2.70-fold). Suberin, like cutin, is a plant cell wall-associated glycerolipid polymer that is found in the root endodermis [67]. Additionally, it was reported that a mutation in *EGL3* (*ENHANCER OF GLABRA 3*) has reduced trichomes and abnormally patterned stomates [68,69]. The downregulation of *EGL3* in *atmin7* (AT1G63650, 2.14-fold) might be related with the defective stomata structure of the mutant plants. Thus, in addition to a putative direct role for AtMIN7 in transport processes required for cutin biosynthesis, changes in the transcriptome of *atmin7* plants may also contribute to the observed cuticle defects and the associated defense phenotypes.

## 3. Discussion

### 3.1. Vesicle Trafficking in Plant Cuticle-Related Component Transport and Cuticle Biosynthesis

Despite many efforts to decipher the mechanisms of cuticle biosynthesis, transport, and regulation, little is known about the transport of the cuticular components to the plant surface. Various lines of evidence indicate that cuticular formation in *Arabidopsis* requires vesicular trafficking [14,15,52,70,71]. Although the related compositions of the vesicles are not well known, they may contain cutin precursors and/or proteins specifically involved in the transport of cuticular-related components to the cell surface. Previous reports have discovered that the trafficking of the intracellular wax components required a GNL1 (gnom like1-1) and ECH (echidna) dependent vesicle trafficking system [15]. It was reported that *gnl1-1* and *ech* mutants of *Arabidopsis* were defective in protein secretions and wax accumulations on the plant surface. GNL1(At5g39500) encodes an ARF-GEF required for ER morphology and vesicle formation from the Golgi apparatus to the ER. Related results also showed that the wax-related components to the plasma membrane requires GNL1-dependent Golgi vesicle trafficking system. Here, our studies firstly demonstrate that AtMIN7, a key component of the vesicle trafficking system and an important immunity-associated protein, is required for the cutin-related component export to the cuticle layer for the related function. The *atmin7* mutant was defective in cuticle formation and the content of the total and several cutin monomer species were significantly reduced in the leaves of the *atmin7* mutant plants compared to WT. However, the wax load did not show significant differences in *atmin7* mutant plants compared to WT plants. Taken together, AtMIN7 might be specifically involved in the export of cutin building blocks or/and key enzymes involved in cutin formation through the intracellular trafficking system.

It is intriguing to find that groups of genes related with lipid transfer proteins (LTPs) and ABC transporters were significantly downregulated in *atmin7* mutant compared to WT plants based on the transcriptome analysis (Supplemental Table S1). Moreover, the hypothetical genes involved in cutin biosynthesis, such as the genes encoding GPATs and GDSL-lipases, were significantly downregulated in *atmin7* mutant compared to WT plants (Table S2). All these indicate that a mutation in *AtMIN7* might not only directly affect the transport of cutin precursors, but also indirectly regulate the expression of proteins and enzymes involved in the deposition of cuticle-related components to the extracellular space.

### 3.2. The Roles of Plant Cuticle During Plant–Pathogen Interaction

The cuticle provides the first line of defense between the plant and its environment. It is becoming increasingly clear that the plant cuticle is an important player in plant–pathogen interactions [7]. Certain cutin monomers or wax components released from the permeable cuticle might be the signals to activate defense responses, or the permeable cuticle could release the defense elicitors from plant hosts, which could activate the faster and stronger plant defense responses [7]. For instance, LACS2 of the *Arabidopsis* plants encodes the long-chain Acyl-CoA synthetase 2. A mutation in *LACS2* resulted in the defective cuticle formation [8]. Researchers found that *lacs2* mutant showed significantly higher resistance against the fungal pathogen *Botrytis cinerea*, which might be due to the induction of the antifungal compounds in the defective cuticle [8]. However, the *lacs2* mutant was more susceptible to *Pto*, indicating that the cuticle distinctly regulates defense against specific pathogens with discrete lifestyles and virulence strategies.

Here, we showed that the *atmin7* plants were more susceptible to the bacterial pathogen *Pto* when plants were infected by spray, but not infiltration. *Pto* proliferates significantly and causes disease following entry into the plant apoplast through natural openings, such as stomata and wounds [45]. In addition to leaf surfaces, cutin also forms a ledge on guard cells of stomata that supports their proper structure and function [43,44]. The *atmin7* mutant plants exhibited impaired stomata and defective stomata cuticular ledge. The differences of bacterial population of the *atmin7* mutant by applying spray and infiltration inoculation assays is consistent with the hypothesis that the impaired stomata cuticular ledge might impair the stomatal-based defense against bacterial entry.

### 3.3. Plant Hormones Are Involved in Cuticle Formation and Cuticle-Mediated Defense Response

Genetic and environmental factors could influence the cuticle quantities and compositions, which suggests that there is an actively regulated process for the cuticle formation [7]. For instance, it was reported that drought stress could enhance the cuticle formation [2]. Additionally, plant hormones play roles in cuticle formation and related functions. For instance, the exogenous application of GA_7_ increases the levels of cuticular lipid compounds and restores the systemic acquired resistance (SAR) in *gl1* mutant plants [72]. Methyl-JA treatment of *Vicia sativa* seedlings increases the *CYP94A1* transcript levels, which is involved in cutin monomer, w-hydroxy fatty acids (FAs) production [50]. Furthermore, the w-hydroxy FAs could induce the barley resistance against fungal pathogen *Erysiphe graminis* f. sp. [73]. ABA plays a positive role in cuticle formation in several plant species to protect plants from water loss during drought stress. A tomato mutation in ABA-deficient *sitiens* (*SIT*)—that is, deficient in ABA production—has a permeable cuticle and increases resistance against *B. cinerea* [48,74]. However, the exact interactions and integrations among the hormones, cuticle, and plant interaction with microbes are still unraveled.

In our study, it was discovered that a mutation in *AtMIN7* led to the reduced levels of hormones, including SA, JA, and ABA. Thus, the *AtMIN7-*dependent vesicle trafficking system might also be associated with the components involved in hormone biosynthesis. Based on our results, these three hormones could specifically rescue the cuticle formation in the leaves of the *atmin7* mutant and restore the surface defense against *Pto*. A possible explanation for these effects of *atmin7* could be that AtMIN7 plays a critical role in the interplay between plant hormones and plant cuticle formation for the related defense function against *Pto* DC3000.

**Figure 8:**
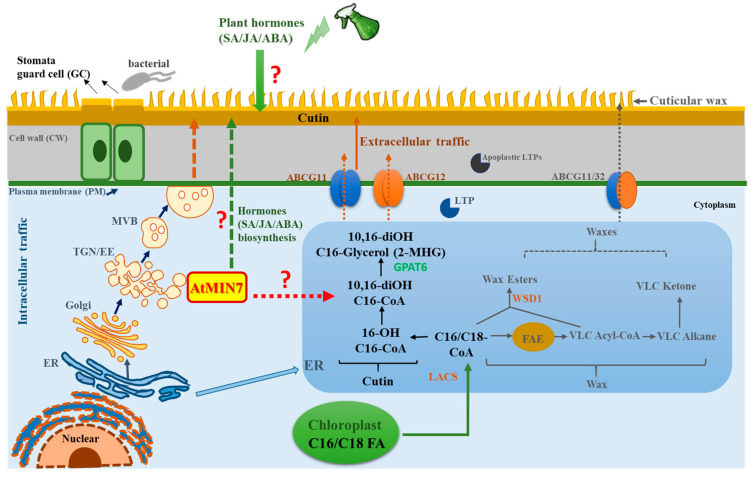
Hypothetical model representing the role of AtMIN7 involved in the cuticle formation and cuticle-associated defense response. The fatty acyl groups (C16/C18 FAs) are generated in the plastid, which are exported from the plastid to the ER as C16/C18-CoA. In the ER, C16/C18-CoA are synthesized as wax and cutin precursors [1,67]. The intracellular transport pathway for wax- and cutin-related components from the ER to the plasma membrane (PM) are predicted by several putative mechanisms, one of which is the Golgi or TGN-mediated secretory pathway [1,13,14,15]. Multivesicular bodies (MVBs) are the late endosomes in plants, which can form intraluminal vesicles involved in the delivery of defense cargoes upon the pathogen infection [75,76]. Here, MVBs might be involved in the intracellular transport of the wax- and cutin-related components. The extracellular export of the wax- and cutin-related components across the PM and cell wall of *Arabidopsis* is predicted to be carried out by the ATP-binding cassette (ABC) transporters and lipid transfer proteins (LTPs) [13,17,52]. However, very limited knowledge was known for the detailed mechanisms. TGN/EE serves as a key sorting station at the intersection of secretory and endocytic pathways [27]. ARF-GEF proteins play a critical role in vesicle budding and vesicular protein sorting [39,40]. In the present study, the TGN/EE-localized AtMIN7, an *Arabidopsis* ARF-GEF, is discovered to be critical for the cutin formation, which may play important roles in the sorting, assembling, or delivering cutin precursors or other related important components, such as the proteins and enzymes, involved in cutin formation through the intracellular trafficking pathway. Moreover, AtMIN7 might be involved in hormones (SA, JA, and ABA) biosynthesis, and also might be associated with the integration between plant hormones (specifically for SA, JA, and ABA) and plant cuticle for the defense against the *Pto* DC3000 pathogen infection in *Arabidopsis* plants. The detailed mechanisms are not well known, which need to be further investigated. FAE: fatty acid elongase; ABCG: ATP-binding cassette (ABC) transporter; LTP: lipid transfer protein; LACS: long-chain acyl-CoA synthetase; ER: endoplasmic reticulum; PM: plasma membrane; TGN/EE: trans-Golgi network/early endosome; MVB: multivesicular body. Solid arrows represent the well-studied mechanisms and pathways currently known. Question marks and dotted arrows indicate the unknown or hypothetical mechanisms and pathways.

In summary, we have discovered that an important vesicle trafficking component, AtMIN7, is involved in cuticle formation, potentially by affecting the transport of cutin precursors and/or cutin biosynthesis-related proteins and enzymes. Notably, these defects correlate with related defense against leaf surface infection with *Pto*. Our model of the mechanism of AtMIN7 activity in cuticle formation is shown in Figure 8. Our study also demonstrates the potential capacity of AtMIN7 to affect hormone biosynthesis and to integrate the response to hormones with modification of the cuticle for defense against *Pto* DC3000. Further investigation of the mechanisms and related networks of AtMIN7 may enable strategies to enhance plant health and yield through the genetic manipulation of plant cuticle, which will reduce the agricultural chemical applications to benefit the environment and human health.

## 4. Materials and Methods

### 4.1. Plant Materials and the Homozygous Line Identification

The seeds of wild-type (WT) Columbia (Col-0) *Arabidopsis* and the *atmin7* mutant line (Salk_013761) were used in this study, which were obtained from the Arabidopsis Biological Resource Center (http://www.arabidopsis.org/). The WT and mutant plants were grown in pots with a commercialized potting mixture in a growth room with the environmental conditions of 60% humidity, 22 °C, 10-h light/14-h darkness photoperiod [77]. The growth condition is also applied for the phenotype observation and pathogen inoculation. The protocol from the Salk Institute Genomic Analysis Laboratory (http://signal.salk.edu/tdnaprimers.2.html) was used to identify the *atmin7* homozygous lines. The homozygous line was identified by the PCR using the primers of the *atmin7* LP and RP, and LBb1.3, which are listed in Supplemental Table S3. The PCR products were analyzed on the agarose gels. The seeds of complementation line of *atmin7* (*35S::MIN7*-GFP) were kindly provided by Dr. Shengyang He [36].

### 4.2. Phylogenetic Tree Construction

Protein sequences of all eight ARF-GEF isoforms were used for multiple protein sequence alignments via Clustal Omega software and for the further phylogenetic analysis (https://www.ebi.ac.uk/Tools/msa/clustalo/). The phylogenetic tree was obtained from Simple Phylogeny via EMBL-EBI. The phylogenetic tree was constructed based on the alignment with the default parameters and displayed in straight branches and cladogram.

### 4.3. Chlorophyll Content Measurement

The four-week-old plant leaves from each genotype were collected and weighted, which were then immersed in 30 mL 80% ethanol in the glass tube at the room temperature. The glass tubes were wrapped in the foil to avoid light exposure. 1 mL of the supernatant was removed at 30 min intervals (up to 120 min) after the immersion. A spectrometer (Thermo scientific Genesys 10S VIS Spectrophotometer, Thermo Scientific^TM^, Columbus, OH, USA) was used for the chlorophyll content measurement with the absorbance at 664 and 647 nm. The micromolar concentration of total chlorophyll per gram of fresh weight of leaf tissues was calculated by the equation: Total micromoles chlorophyll = 7.93(A664) +19.53(A647) [42].

### 4.4. Plant Hormone Treatments

Hormone treatments were conducted by spraying SA (1 mM), JA (50 µM), ABA (50 µM), gibberellic acid (GA_3_, 100 µM), and indole-3-acetic acid (IAA,10µM) solutions to the *Arabidopsis* plant leaves until there was imminent runoff, which indicated that the leaf surfaces were well covered with the hormone solution (generally, 5 mL solution for each plant). For hormone treatment solution preparation, 100% EtOH (500 µL) was firstly used to dissolve SA, JA, and ABA, which were further diluted in 1 L distilled and deionized water to reach the final treatment concentration, respectively. Accordingly, 1 L distilled and deionized water with 500 µL 100% EtOH was sprayed as the control [72,78,79,80]. During the spray process, a spray bottle with a nozzle set was used to spray a very fine mist [72,81]. 24 h after the hormone treatments, the plant leaves were subjected to the further tests including the staining with TB or surface inoculation with *Pto* DC3000.

### 4.5. Pathogen Culture Condition and Bacterial Growth Assays

In this study, the *Pto* DC3000 cells were grown on the King’s B plate with rifampin as the selective antibiotic. The fresh bacterial cells grown on plates were collected and diluted in 10 mM MgCl_2_. For the pathogen inoculation assay, bacterial broth with the concentration of 1 × 10^5^ CFU (colony forming unit)/mL (OD600 = 0.0002) in 10 mM MgCl_2_ were infiltrated into the abaxial side of the four-week-old *Arabidopsis* leaves using the 1 mL syringe. The residues were wiped off from the leaves after infiltration. The inoculated plants were left for one hour and then returned to the growth room. The bacterial growth was determined three days after the inoculation [9]. Three leaf discs for each technical replicate were collected. For each treatment, six technical replicates were applied. The bacterial titer was determined by grinding leaf discs thoroughly in 10 mM MgCl_2_ with a serial of dilution. The final diluted solution was plated onto the King’s B plates with rifampin. Colonies were counted and used to calculate the mean CFU/cm^2^ for each treatment, and the final values were log transformed. Each biological replicate was calculated as a single data point. Multiple independent biological replicates were used to calculate the mean, SD, and significant differences.

### 4.6. Cuticular Wax and Cutin Content Analysis

The cuticular wax and cutin monomer contents of leaves from four-week-old plants were determined as described by Lü and Yang et al. [2,82,83]. For wax extraction, four-week-old *Arabidopsis* rosette leaves from each genotype were weighted and extended gently for the area measurement. The collected leaves were submerged in 20 mL hexane twice for 30 s. The solutions were mixed into a clean vial for the total cuticular extraction. 50 µL internal standard (ISTD, N-Hexadecane, ICN Bio-medicals Inc., concentration 5 µg/50 µL) was added into the vials by a micropipette. After the addition of ISTD, the samples were dried up under the evaporating unit (N^2^ gas). Then, the samples were derivatized with 50 µL of BSTFA (N,O-Bis(trimethylsilyl)trifluoroacetamide) and immediately placed in a 100 °C dry bath for 15 min. 200 µL hexane was added into each sample to dissolve the wax extracts. Agilent 7820A gas chromatograph equipped with a flame ionization detector, together with a 30-m, 0.25-mm DB-5 capillary column with helium as the carrier gas, were used for GC analysis. The column temperature was programmed as following: an initial temperature of 80 °C for two minutes, increased to 200 °C (40 °C min^−1^) and held for two minutes, then increased to 270 °C (10 °C min^−1^) and held for two minutes, and finally increased to 320 °C (3 °C min^−1^) and held for 40 min. The flame ionization detector (FID) peak areas relative to the internal standard n-Hexadecane was applied to quantify the samples. An Agilent 7890A gas chromatograph equipped with an Agilent 5975C mass spectrometric detector was applied to determine the individual wax species (70 eV; mass-to-charge ratio of 50–750) [82].

The left plant leaf samples used for the wax extraction were applied for the further cutin monomer extraction, which were immersed in 15 mL isopropanol in an 85 °C incubator for 10–15 min, and then placed on a shaker with stirring for two hours. Furthermore, the samples were delipidated deeply with isopropanol, chloroform/methanol (2:1, *v*/*v*), chloroform/methanol (1:2, *v*/*v*), and methanol for one day. Finally, the tubes with samples were centrifuged for 10 min at 2000 rpm. The solvent was removed from each tube. The residues in the tubes were dried up under the hood for three to five days until the constant weight was reached. Next, the dried samples were weighed and undergone depolymerization, which is the methanolysis process with sodium methoxide. 25 mg methyl heptadecanoate and 25 mg ω-pentadecalactone were applied as internal standards. Samples with internal standards, 0.9 mL methyl acetate, 1.5 mL sodium methoxide, and 3.6 mL methanol were heated at 60 °C for two hours. After cooling down, 10 mL methylene dichloride and 0.5 mL glacial acetic acid were used for fatty acid methyl esters extraction. After this step, the extracts were washed with 0.5 M NaCl for three times, and then dried up under N^2^ gas. For the further derivatization, the hydroxyl groups were acetylated by 200 μL pyridine/acetic anhydride (1:1, *v*/*v*) at 60 °C for one hour, to produce the corresponding acetyl derivatives. The extracts were evaporated under N^2^ gas, and dissolved in chloroform, which were ready for GC–MS analysis. Agilent 7820A gas chromatograph equipped with a flame ionization detector, together with a 30-m, 0.25-mm DB-5 capillary column with helium as the carrier gas, were used for the sample analysis. The column temperature was programmed as following: an initial temperature of 80 °C increased to 200 °C (15 °C min^−1^), then increased to 230 °C (2 °C min^−1^), held for 10 min. The FID peak areas relative to the internal standard methyl heptadecanoate and ω-pentadecalactone were applied to quantify the samples. The individual cutin monomers were measured with an Agilent 7890A gas chromatograph equipped with an Agilent 5975C mass spectrometric detector (70 eV; mass-to-charge ratio of 50–750) [2,82].

### 4.7. SEM and TEM

Hitachi H-7500 TEM equipped with the SIA-L12C digital camera was used to observe the cuticle layer of the leaves and cuticular ledge of stomata. Hitachi Schottky field emission SU5000 SEM equipped with the Bruker Energy Dispersive X-ray Spectrometer ESPRIT system for the qualitative and quantitative elemental microanalysis was used to observe the leaf surface, leaf section, and stomata structure. Leaf samples were collected from plants at four weeks of growth. Samples were prepared and observed at the Molecular & Cellular Imaging Center (MCIC) at The Ohio State University as previously reported [84,85].

### 4.8. Hormone Level Determination

The methods of hormone SA, JA, and ABA extraction and detection were modified from the papers published by Forcat et al (2008) [86] The leaves from four-week-old *Arabidopsis* plants (approximately 110 mg fresh weight per sample) were collected with 400 μL extraction buffer containing 10% methanol and 1% acetic acid. The isotope labelled internal standards (d_4_-SA, d_5_-JA, and d_6_-ABA) were added at the beginning of the extraction. The amounts of the internal standards were 15 ng ^2^H4-SA (d_4_-SA, CDN Isotopes, Pointe-Claire, QC, Canada, part #: D-1156) and 150 ng ^2^H5-JA (d_5_-JA, CDN Isotopes, Pointe-Claire, QC, Canada, part #D-6936), and 1 ng of ^2^H6-ABA (d_6_-ABA, Toronto Research Chemicals, North York, ON, USA; part #: A110002). After the addition of the extraction buffer, the tubes with extracts were incubated on ice for 30 min, and then centrifuged at 4 °C (13,000× *g*, 10 min). The supernatant was collected into new tubes. The left pellets were re-extracted with 400 μL extraction buffer without adding the internal standard. The tubes with extracts were centrifuged again. The two supernatant solutions were mixed for further analysis through UPLC/ESI/MS by the Thermal Fisher Ultimate 3000 system (Thermal Fisher, Waltham, MA, USA) with a 3 μm C18 (100 mm × 2.0 mm) column (Waters Company, Milford, MA, USA) at 35 °C. The mobile phase was set for a continuum gradient from (94.9% H_2_O: 5% CH_3_CN: 0.1% CHOOH) to (5% H_2_O: 94.9% CH_3_CN: 0.1% CHOOH) for about 20 min. Multiple reaction monitoring (MRM) of ion pairs for the labelled and endogenous hormones were used for the analysis of the compounds. The retention time and mass transitions for SA, JA, and ABA were verified based on the authentic standards. The daughter masses were indicated in the brackets listed below. The transition settings for SA, JA, and ABA: ^2^H_4_-SA 141 (97), SA 137 (93), SA-Gly 299 (93), ^2^H_5_-JA 214 (61), JA 209 (59), JA-Ile/Leu 322 (130), ^2^H_6_-ABA 269 (159), and ABA 263 (153).

### 4.9. RNA-Seq Data Analysis

The total RNA was extracted from the leaves of four-week old WT Col-0 and *min7* mutant plants using TRIzol Reagent (Invitrogen, Carlsbad, MA, USA) following the manufacturer’s instructions. Total RNA was quantified and checked using a Nanodrop-ND 8000 spectrophotometer (Thermo Fisher Scientific, Columbus, OH, USA). Replicated samples with the RNA integrity number of 7.5 and above were chosen for the further analysis. The RNA-Seq was further carried out with an Illumina HiSeq4000 PE150 at Novogene Bioinformatics Technology Co., Ltd. (Beijing, China). The clean data were mapped to the Tair10 genome release files using TopHat2 (mismatch = 2). The read numbers mapped to each gene were counted using HTSeq (-m union) and the reads count was used to calculate the FPKM (expected number of fragments per kilobase of transcript sequence per million base pairs sequenced) [87]. The DEGSeq R package (*q* < 0.005 and log2 (fold change) > 2) was used for the differential gene expression analysis. The *p*-values were adjusted with the Benjamini and Hochberg method.

## Supplementary Materials

Supplementary materials can be found at https://www.mdpi.com/1422-0067/21/15/5547/s1.
