# Supplementary material for: Deciphering the Novel Role of AtMIN7 in Cuticle Formation and Defense against the Bacterial Pathogen Infection
Source: Int J Mol Sci. 2020 Aug 3;21(15):5547. doi: 10.3390/ijms21155547 (PMC7432873; doi:10.3390/ijms21155547)
Supplement: Supplementary file 1 [file ijms-21-05547-s001.zip › MIN7 Supplemental Material IJMS 08022020/MIN7 Supplemental Material Intl.J.Mol.Sci 07152020 .docx]

Article

Deciphering The Novel Role of AtMIN7 in Cuticle Formation and Defense Against The Bacterial Pathogen Infection

Supplemental material

Supplemental Figure S1


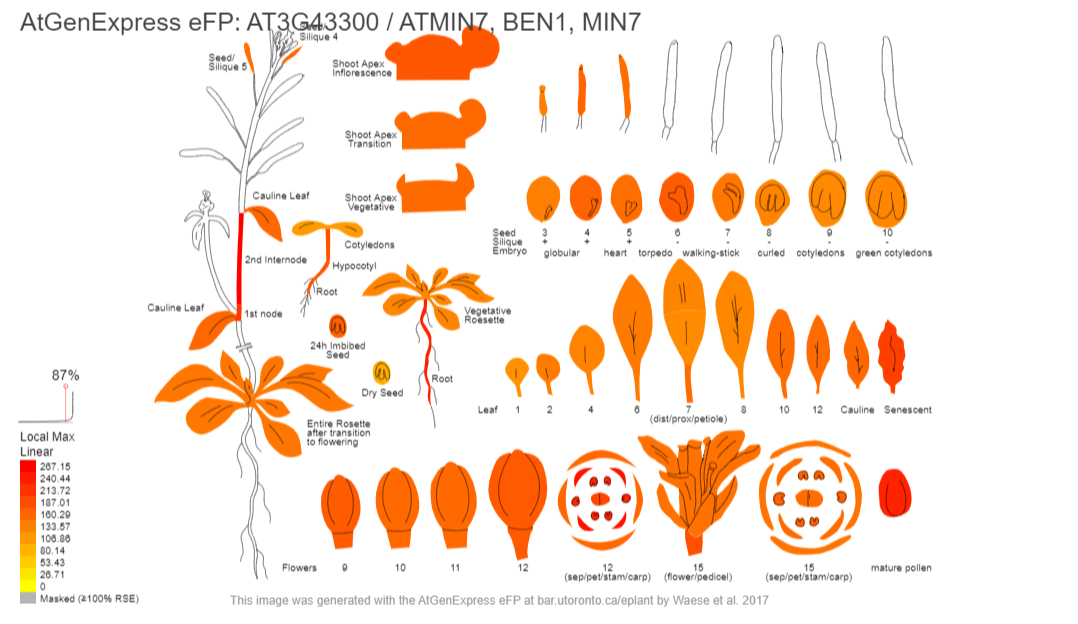


**Supplemental Figure S1 The *AtMIN7* gene expressions in different tissues of Arabidopsis plants.** *AtMIN7* gene expression at different tissues of Arabidopsis plant predicted by Arabidopsis eFP Browser. <http://bar.utoronto.ca/eplant/>.

Supplemental Figure S2

**
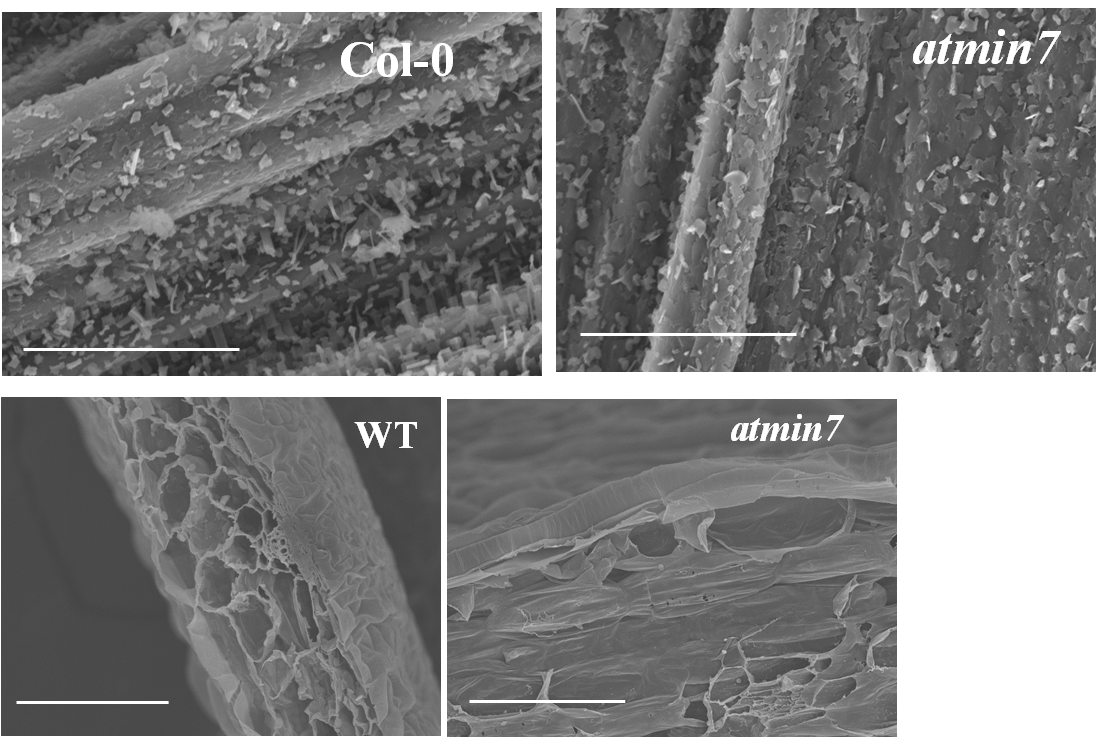
**

**Supplemental Figure S2. SEM images of sections of wild type (WT) Col-0 and *atmin7* leaves** (Scale bars, 100 µm).


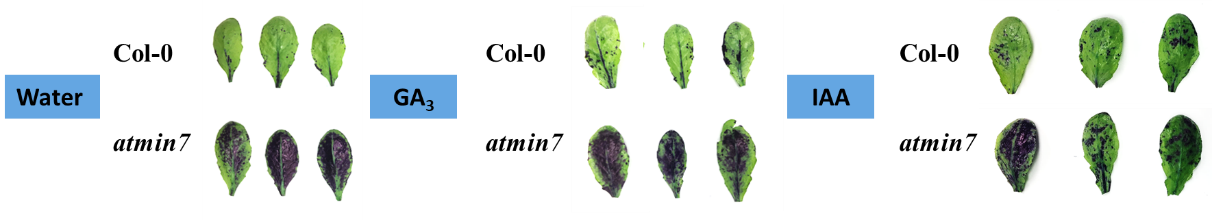
 Supplemental Figure S3

**Supplemental Figure S3. Plant hormones play important roles in cuticle formation in *atmin7* mutant plants.** Leaves of 4-week-old WT or *atmin7* plants were stained with TB 24 hours after the spraying with water, Gibberellic acid (GA_3_, 100µM), Indole-3-acetic acid (IAA) (10µM). 100% EtOH (500µl) was firstly used to dissolve SA, JA, and ABA, which were then further diluted in 1L distilled, deionized water to reach the final treatment concentration, respectively. Accordingly, 1L distilled and deionized water with an equivalent amount of 100% EtOH (500µl) was prepared to spray as the control.

**Supplemental Table S1. Excel form of genes whose transcript abundance is down-regulated in *atmin7* leaves compared to WT Col-0 plants by imposing the stringent cut-off criteria (P-value of <0.05 and |log2 fold change (FC)|≥ 2)**

**Supplemental Table S2. Summary of Arabidopsis genes hypothetically implicated in cutin biosynthesis with the reduced expressions in *atmin7* mutant plants compared to WT Col-0 plants.** The log2 FC indicate the extent of differential gene expression ([*atmin7*] vs [Col-0]). FC represents Fold Change.

| **class** | **GENE** | **ID** | **FUNCTION** | **log2 fC** |
| --- | --- | --- | --- | --- |
|  | **LTP4** | **AT5G59310** | **Lipid transfer protein** | **-5.45** |
|  | **LTP3** | **AT5G59320** | **Lipid transfer protein** | **-4.62** |
|  | **LTP6** | **AT3G08770** | **Lipid transfer protein** | **-3.19** |
| **LTP/LTPG** | **LTP2** | **AT2G38530** | **Lipid transfer protein** | **-3.12** |
|  | **LTP** | **AT1G05450** | **Lipid transfer protein** | **-4.51** |
|  | **LTPG15** | **AT2G48130** | **Lipid transfer protein** | **-3.50** |
|  | **LTPG5** | **AT3G22600** | **Lipid transfer protein** | **-3.02** |
|  | **LTP** | **AT2G37870** | **Lipid transfer protein** | **-2.50** |
|  | **UMAMIT8** | **AT4G16620** | **nodulin MtN21-like transporter** | **-2.69** |
|  | **UMAMIT29** | **AT4G01430** | **plasma membrane-localized amino acid transporter** | **-2.21** |
| **TRANSPORTER** | **UMAMIT33** | **AT4G28040** | **nodulin MtN21-like transporter** | **-2.11** |
|  | **UMAMIT28** | **AT1G01070** | **plasma membrane-localized amino acid transporter** | **-2.10** |
| **ABCG** | **ABCG6** | **AT5G13580** | **ATP-BINDING CASSETTE G6** | **-2.1** |
|  | **MYB41** | **AT4G28110** | **MYB domain protein** | **-4.42** |
|  | **MYB114** | **AT1G66380** | **MYB domain protein** | **-4.67** |
|  | **MYB49** | **AT5G54230** | **MYB domain protein** | **-4.42** |
| **Myb tf** | **MYB17** | **AT3G61250** | **MYB domain protein** | **-2.80** |
|  | **MYB75** | **AT1G56650** | **MYB domain protein** | **-2.29** |
| **Cutin Synthase** | **NA** | **AT3G50400** | **GDSL-motif esterase/acyltransferase/lipase.** | **-5.68** |
|  | **NA** | **AT2G23540** | **GDSL-motif esterase/acyltransferase/lipase.** | **-3.00** |
| **GPAT** | **GPAT7** | **AT5G06090** | **glycerol-3-phosphate acyltransferase 7** | **-4.68** |
|  | **GPAT5** | **AT3G11430** | **glycerol-3-phosphate acyltransferase 5** | **-3.8** |
| **suberin synthesis** | **RWP1** | **AT5G41040** | **feruloyl-CoA transferase for suberin synthesis** | **-2.7** |
| **Transcription Factor** | **EGL3** | **AT1G63650** | **Mutant has reduced trichomes, anthocyanin, and seed coat mucilage and abnormally patterned stomates.** | **-2.14** |

**Supplemental Table S3. Primers used in this study.**

| **Name** | **Sequence 5' to 3'** | **Purpose** |
| --- | --- | --- |
| **Atmin7 LP** | **TGGAAAGTGAAATTGGTGAGC** | **atmin7 T-DNA line genotyping** |
| **Atmin7 RP** | **CAAGGATTCTTCTCTGCATGG** |  |
| **LBb1.3** | **ATTTTGCCGATTTCGGAAC** | **T-DNA left border primer** |
| **AtMIN7-qF** | **GCATGGATCAAACCAGATCTCC** | **QRT-PCR for *AtMIN7* expression pattern** |
| **AtMIN7-qR** | **GGCAGGAGACTGCTTCAATTCTTC** | **QRT-PCR for *AtMIN7* expression pattern** |
